# Supplementary material for: The translesion polymerase Pol Y1 is a constitutive component of the B. subtilis replication machinery
Source: Nucleic Acids Res. 2024 Jul 25;52(16):9613–29. doi: 10.1093/nar/gkae637 (PMC11381352; doi:10.1093/nar/gkae637)
Supplement: gkae637_Supplemental_File [file gkae637_supplemental_file.pdf]

“The Translesion Polymerase Pol Y1 is a Constitutive Component of the *B. subtilis* Replication Machinery”

McKayla E. Marrin[1], Michael R. Foster[1], Chloe M. Santana[1], Yoonhee Choi[1], Avtar S. Jassal[1], Sarah J. Rancic[1], Carolyn R. Greenwald[1], Madeline N. Drucker[1], Denholm T. Feldman[1], Elizabeth S. Thrall[1**]

[1]Department of Chemistry and Biochemistry, Fordham University, Bronx, NY, 10458, USA

[**]Correspondence: Tel: +1 718-817-4495; Fax: +1 718-817-4432; Email: ethrall@fordham.edu

SUPPLEMENTARY INFORMATION

This PDF file includes:

Figures S1 to S8
Tables S1 to S9
Supplementary Methods
Supplementary References

Supplementary Figures

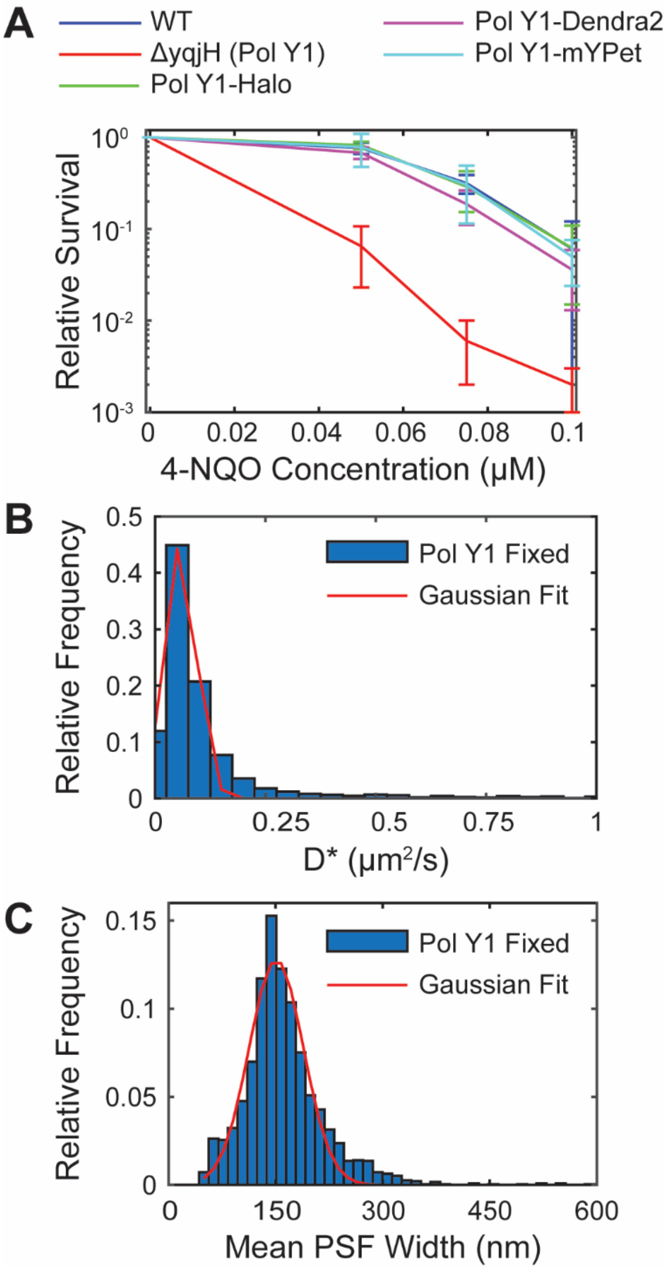

Figure S1. (A) Relative survival of *B. subtilis* strains treated with different concentrations of 4-NQO: WT Pol Y1, Pol Y1 knockout, Pol Y1-Halo fusion, Pol Y1-Dendra2 fusion, and Pol Y1-mYPet fusion strains. (B) Distribution of the Pol Y1 apparent diffusion coefficient D^* measured in fixed cells and the corresponding Gaussian fit. (C) Distribution of the mean point spread function (PSF) width for static trajectories recorded with a long 250 ms integration time and the corresponding Gaussian fit.

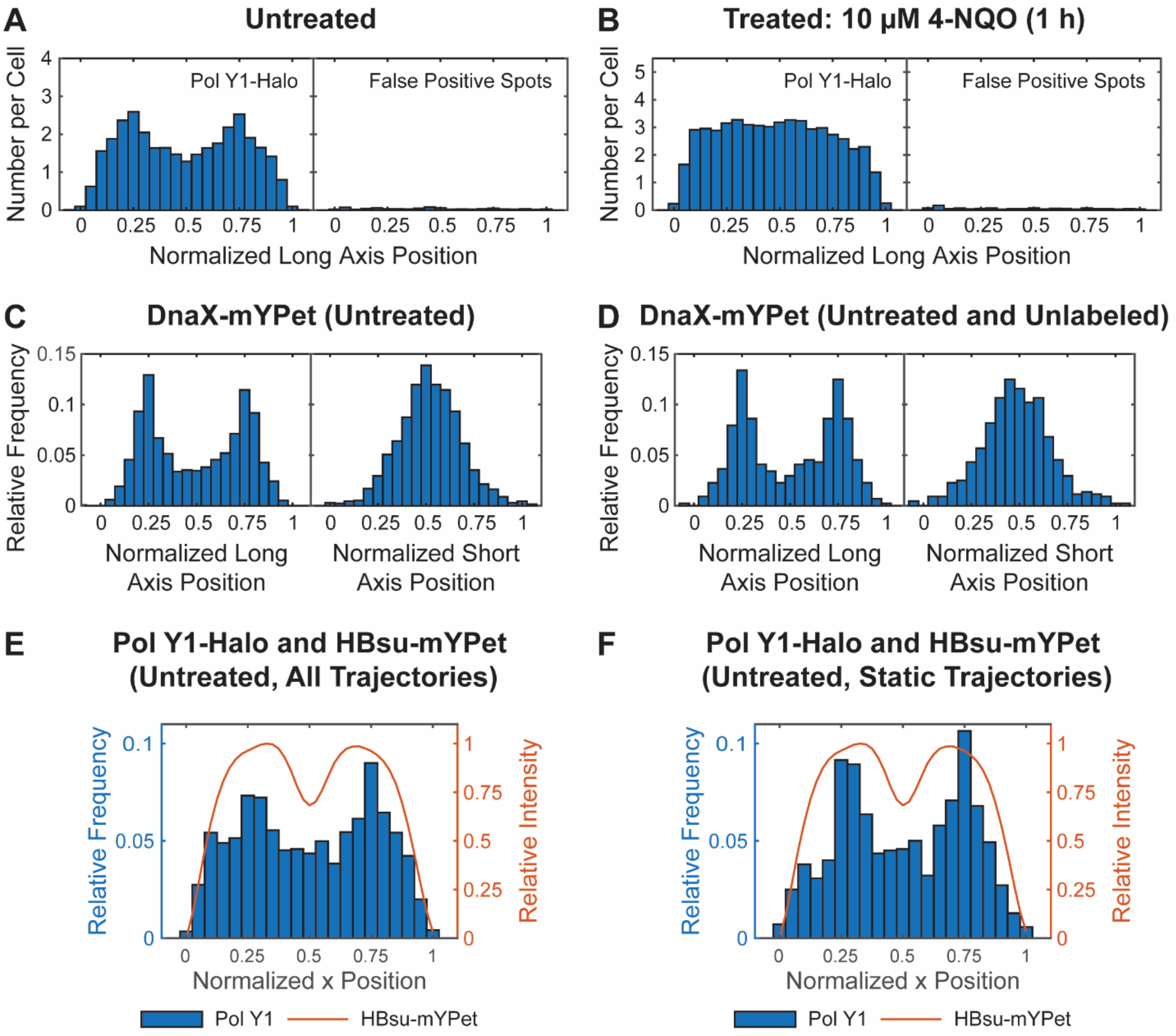

Figure S2. Cellular localization of DnaX-mYPet, Pol Y1-Halo, and the nucleoid label HBsu-mYPet. Long cell axis projections of Pol Y1 (left) and false positive spots (right) in (A) untreated cells and (B) cells treated with 10 μM 4-NQO for 1 h on a per cell basis. Long and short cell axis projections of DnaX in untreated cells either (C) labeled with 2.5 nM JFX_{554} or (D) unlabeled. Long cell axis projections of the nucleoid label HBsu and (E) all Pol Y1 trajectories or (F) static Pol Y1 trajectories ($D^* < 0.14$ μm^2/s) in untreated cells. Panel (C) is reproduced from Figure 3B.

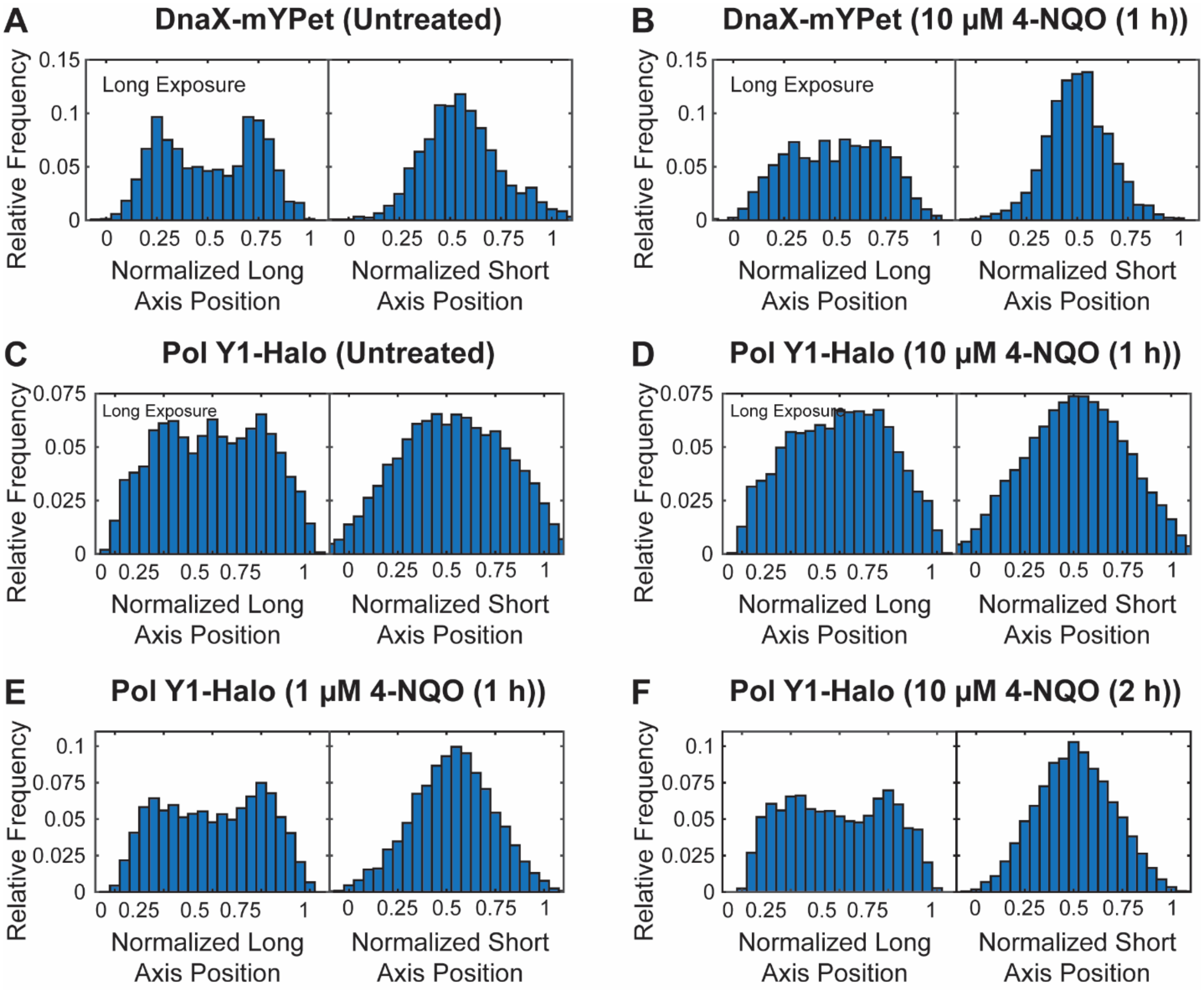

Figure S3. Cellular localization of DnaX-mYPet and Pol Y1-Halo. Long and short cell axis projections of (A, B) DnaX and (C, D) Pol Y1 in untreated cells and cells treated with 10 µM 4-NQO for 1 h, respectively, recorded with a long 250 ms integration time. Long and short cell axis projections of Pol Y1 in cells treated with (E) 1 µM 4-NQO for 1 h and (F) 10 µM 4-NQO for 2 h.

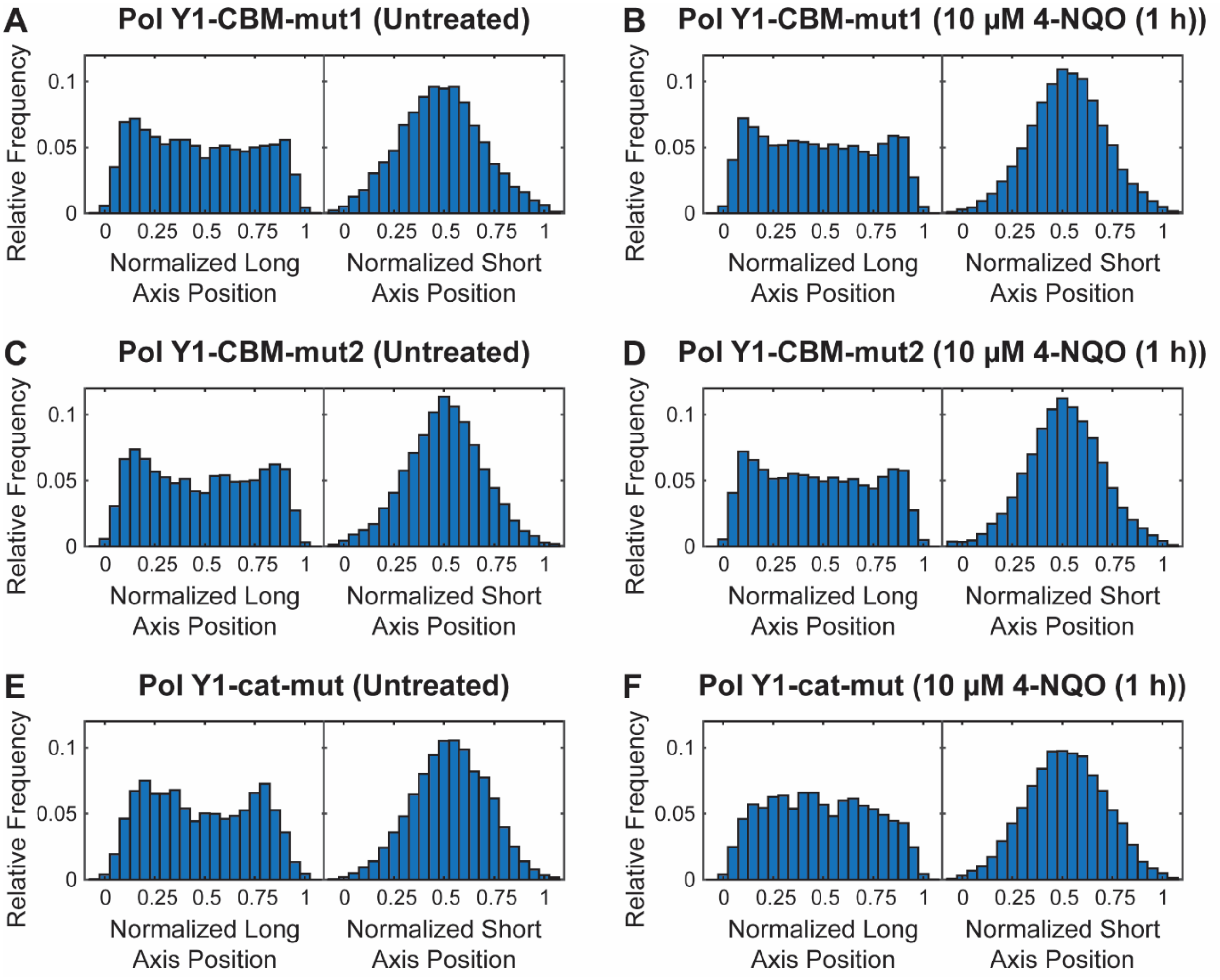

Figure S4. Cellular localization of Pol Y1-Halo mutants. Long and short cell axis projections of Pol Y1-CBM-mut1, Pol Y1-CBM-mut2, and Pol Y1-cat-mut in (A, C, E) untreated cells and (B, D, F) cells treated with 10 μM 4-NQO for 1 h, respectively.

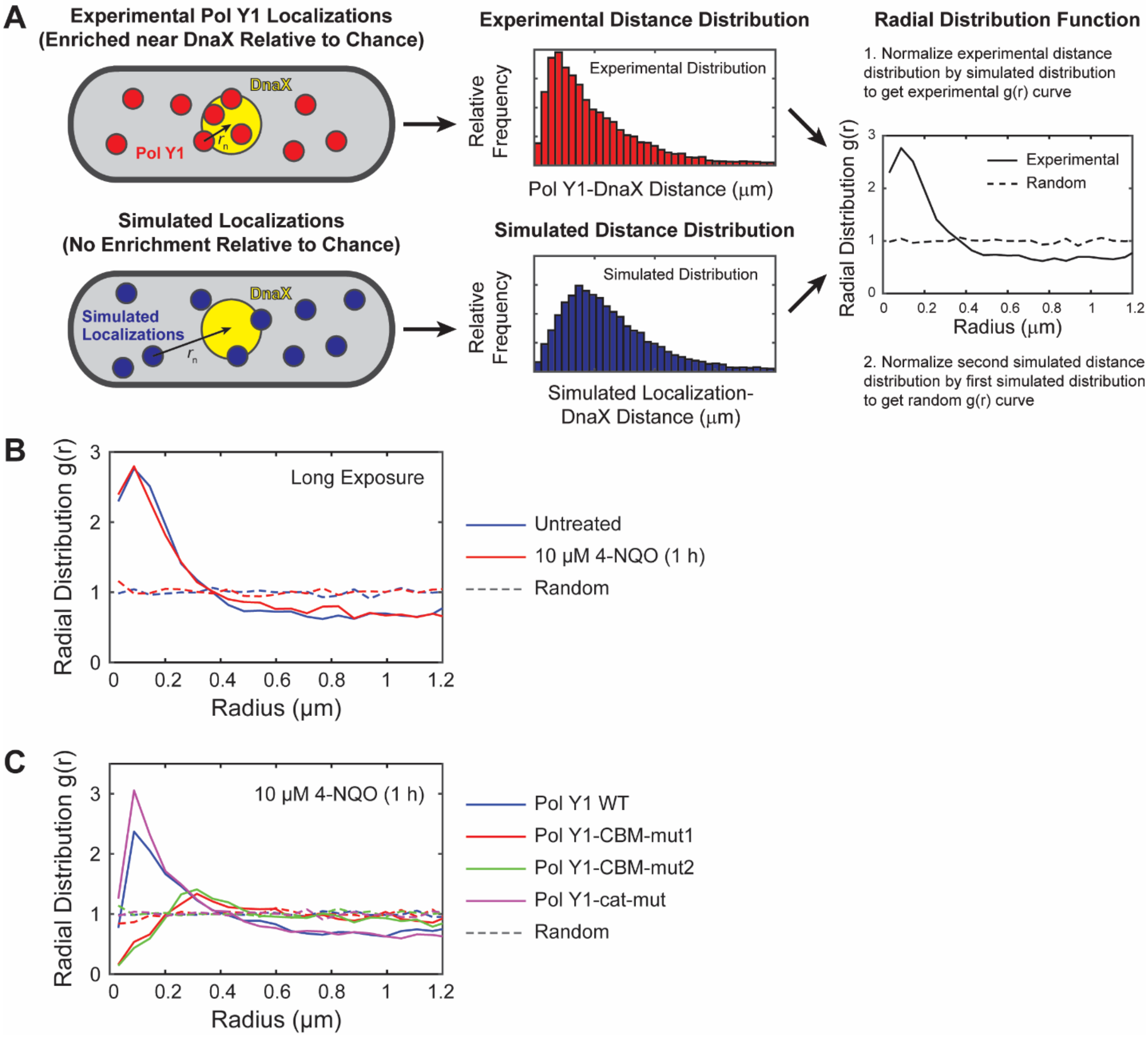

Figure S5. Radial distribution function $g(r)$ analysis of Pol Y1-Halo and DnaX-mYPet colocalization. (A) Cartoon of radial distribution function $g(r)$ analysis. (B) Pol Y1-DnaX $g(r)$ in untreated cells and cells treated with 10 μM 4-NQO for 1 h recorded with a long 250 ms integration time. (C) Pol Y1-DnaX $g(r)$ for WT Pol Y1, Pol Y1-CBM-mut1, Pol Y2-CBM-mut2, and Pol Y1-cat-mut in cells treated with 10 μM 4-NQO for 1 h. Random $g(r)$ curves in (B) and (C) are shown as dashed lines.

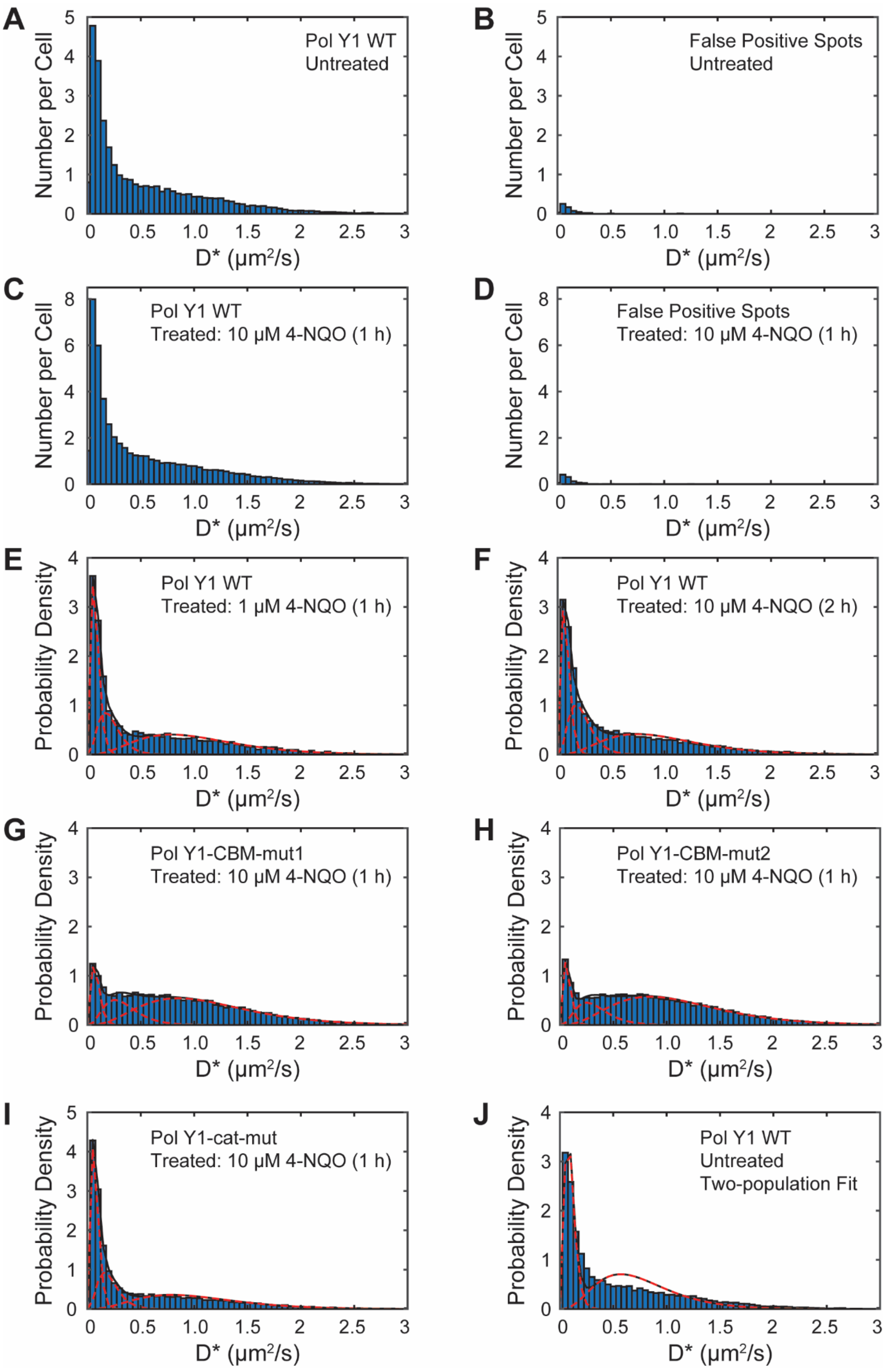
A
Pol Y1 WT
Untreated
Number per Cell
D* (μm²/s)
B
False Positive Spots
Untreated
Number per Cell
D* (μm²/s)
C
Pol Y1 WT
Treated: 10 μM 4-NQO (1 h)
Number per Cell
D* (μm²/s)
D
False Positive Spots
Treated: 10 μM 4-NQO (1 h)
Number per Cell
D* (μm²/s)
E
Pol Y1 WT
Treated: 1 μM 4-NQO (1 h)
Probability Density
D* (μm²/s)
F
Pol Y1 WT
Treated: 10 μM 4-NQO (2 h)
Probability Density
D* (μm²/s)
G
Pol Y1-CBM-mut1
Treated: 10 μM 4-NQO (1 h)
Probability Density
D* (μm²/s)
H
Pol Y1-CBM-mut2
Treated: 10 μM 4-NQO (1 h)
Probability Density
D* (μm²/s)
I
Pol Y1-cat-mut
Treated: 10 μM 4-NQO (1 h)
Probability Density
D* (μm²/s)
J
Pol Y1 WT
Untreated
Two-population Fit
Probability Density
D* (μm²/s)

Figure S6. Apparent diffusion coefficient (D^*) distributions for Pol Y1-Halo and corresponding three-population fits. D^* distributions for WT Pol Y1 (A, C) and false positive spots (B, D) in untreated cells and cells treated with 10 μM 4-NQO for 1 h, respectively, on a per cell basis. D^* distributions for WT Pol Y1 in cells treated with (E) 1 μM 4-NQO for 1 h and (F) 10 μM 4-NQO for 2 h. D* distributions for (G) Pol Y1-CBM-mut1, (H) Pol Y1-CBM-mut2, and (I) Pol Y1-cat-mut mutants in cells treated with 10 μM 4-NQO for 1 h. (J) D^* distributions for WT Pol Y1 in untreated cells with a two-population instead of three-population fit.

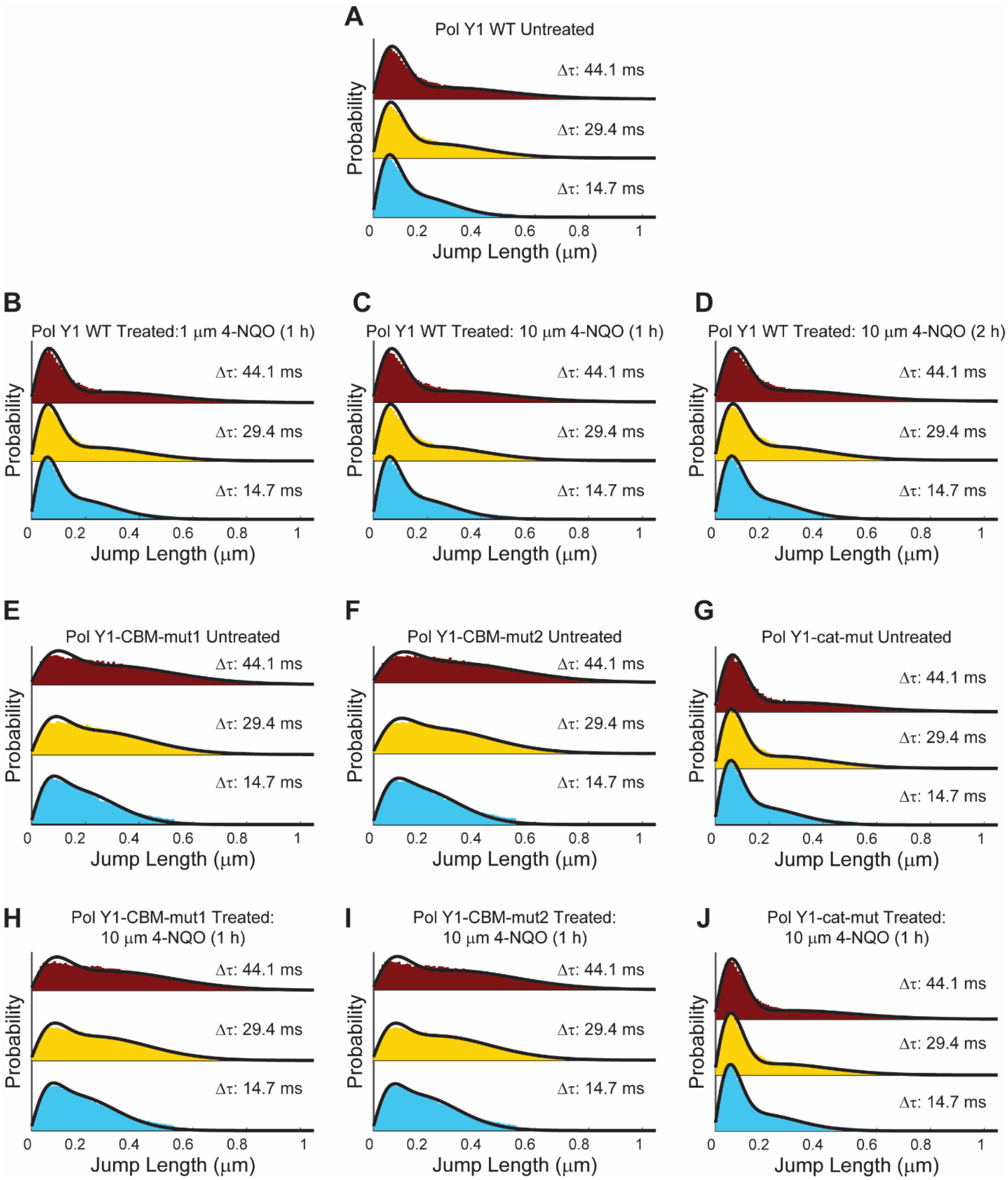

Figure S7. Spot-On diffusion analysis for Pol Y1-Halo, showing jump length distributions and corresponding two-population fits. WT Pol Y1 in (A) untreated cells and cells treated with (B) 1 μM 4-NQO for 1 h, (C) 10 μM 4-NQO for 1 h, or (D) 10 μM 4-NQO for 2 h. (E, H) Pol Y1-CBM-mut1, (F, I) Pol Y1-CBM-mut2, and (G, J) Pol Y1-cat-mut mutants in untreated cells and cells treated with 10 μM 4-NQO for 1 h respectively.

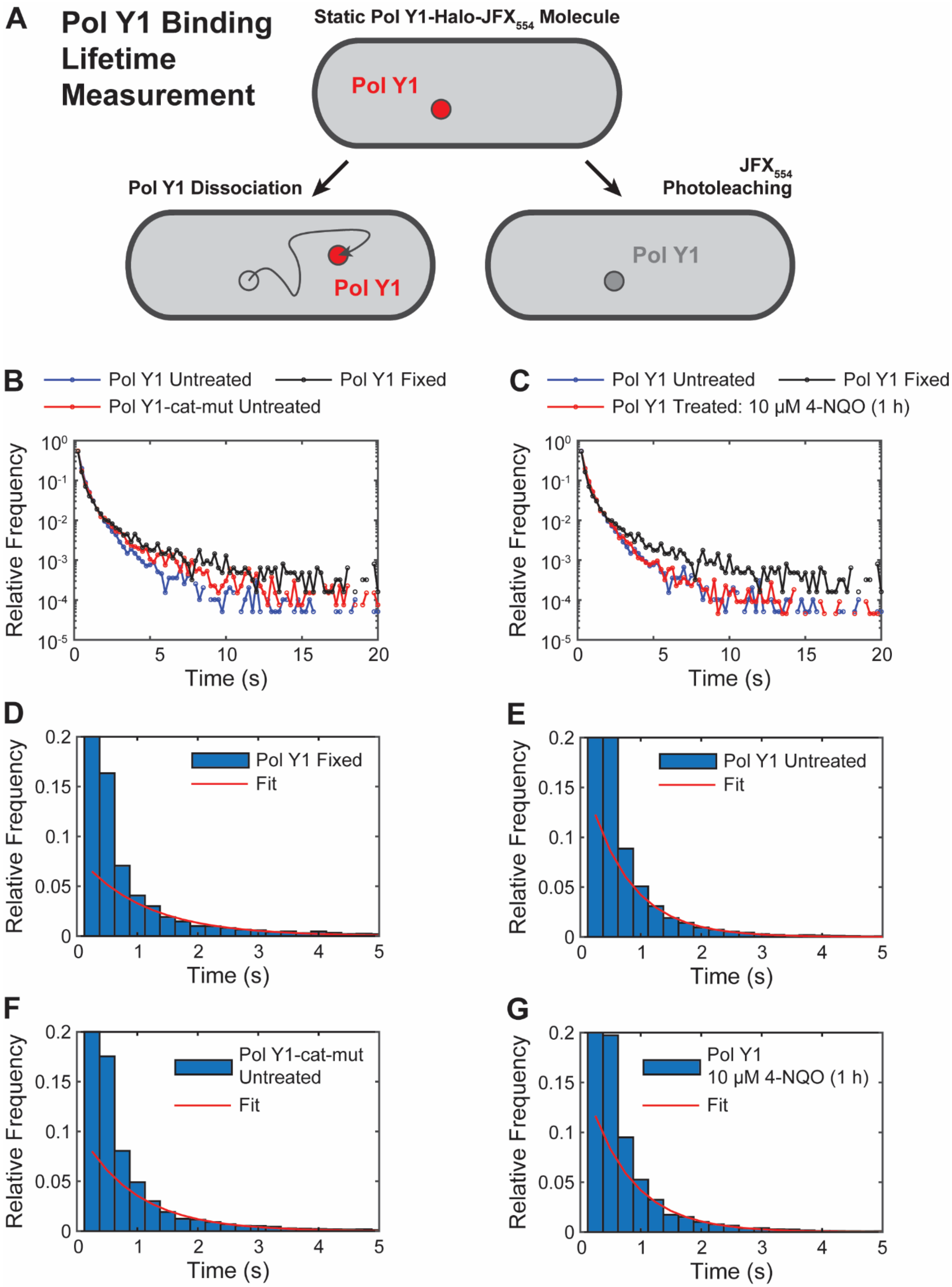
A
Pol Y1 Binding Lifetime Measurement
Static Pol Y1-Halo-JFX554 Molecule
Pol Y1
Pol Y1 Dissociation
JFX554 Photoleaching
Pol Y1
Pol Y1
B
Pol Y1 Untreated
Pol Y1 Fixed
Pol Y1-cat-mut Untreated
Relative Frequency
Time (s)
C
Pol Y1 Untreated
Pol Y1 Fixed
Pol Y1 Treated: 10 μM 4-NQO (1 h)
Relative Frequency
Time (s)
D
Pol Y1 Fixed
Fit
Relative Frequency
Time (s)
E
Pol Y1 Untreated
Fit
Relative Frequency
Time (s)
F
Pol Y1-cat-mut Untreated
Fit
Relative Frequency
Time (s)
G
Pol Y1 10 μM 4-NQO (1 h)
Fit
Relative Frequency
Time (s)

Figure S8. Pol Y1-Halo binding lifetime measurements. (A) Cartoon of Pol Y1 dissociation and JFX_{554} photobleaching pathways. (B) Apparent Pol Y1 binding lifetime for WT Pol Y1 in untreated cells, Pol Y1-cat-mut in untreated cells, and WT Pol Y1 in cells fixed with formaldehyde. (C) Apparent Pol Y1 binding lifetime for WT Pol Y1 in untreated cells, cells treated with 10 μM 4-NQO for 1 h, and cells fixed with formaldehyde. Distributions of apparent binding lifetime and corresponding exponential fits for (D) WT Pol Y1 in fixed cells, (E) WT Pol Y1 in untreated cells, (F) Pol Y1-cat-mut in untreated cells, and (G) WT Pol Y1 in cells treated with 10 μM 4-NQO for 1 h. (Note that the *y*-axes are truncated in D – G to show the longer timescale behavior more clearly.)

Supplementary Tables

Table S1: Oligonucleotides used in this study

Number	Designation	Sequence (5′-3′)
oEST008	loxP ab rev univ short	GACCAGGGAGCACTGGTCAAC
oEST023	yqjH-amplify-for	GCGGGTTGATATTATGCTCCTG
oEST028	yqjH-downstream-rev	CGACAACTTCAGATGGGCTGGTGTTC
oEST029	yqjH-amplify-rev-nostop	GCTTTTCTTTTCATCTTGAA
oEST030	yqjH-linker-halo-for	ttcaagatgaaaagaaaagcGGCTCTGGACAGGGCTCAGG
oEST031	halo-spec-rev	gcgagggagcagaaggatccTTACTAGCCGCTGATTTCTA
oEST032	spec-for	GGATCCTTCTGCTCCCTCGCT
oEST033	yqjH-linker-dendra2-for	ttcaagatgaaaagaaaagcCTCGAGGGATCTGGCGGATC
oEST034	dendra2-spec-rev	gcgagggagcagaaggatccCTACCAGACTTGTGACGGCA
oEST035	yqjH-linker-mYPet-for	ttcaagatgaaaagaaaagcCTCGAGGGATCAGGACAGGG
oEST036	mYPet-spec-rev	gcgagggagcagaaggatccTTACTTGTAAAGTTCATTCA
oEST037	yqjH-upstream-for	CATCAGTCACCGTATTGACT
oEST038	yqjH-Nter-rev	TCGGCTCTTTCCCGGCATAA
oEST039	yqjH-Nter-spec-iso-for	ttatgccgggaaagagccgaGGATCCTTCTGCTCCCTCGC
oEST040	yqjH-Cter-spec-iso-rev	attcagcttttcttttcatcGACCAGGGAGCACTGGTCAA
oEST041	yqjH-Cter-for	GATGAAAAGAAAAGCTGAATCGC
oEST048	yqjW-downstream-rev	GTTCATCAAATTGGCTCACG
oEST049	yqjW-upstream-for	AATATAAATCGGCCGGCCAG
oEST058	yqjH-CBM-mut1-rev	TGAACgcATCGgcCTGTTTATAGGCCTGCTCTTTTTCTAC TAAATCCG
oEST059	yqjH-CBM-mut1-for	CCTATAAACAGgcCGATgcGTTCAGCTTTAATGAAGATGC GAAGGAT
oEST060	yqjH-CBM-mut2-rev	TGgcCAAATCGAGCgcTTTATAGGCCTGCTCTTTTTCTAC TAAATCCG
oEST061	yqjH-CBM-mut2-for	CCTATAAAgcGCTCGATTTGgcCAGCTTTAATGAAGATGC GAAGGAT
oEST062	yqjH-cat-mut-rev	CCATATAGCCTgCGgCGATGGAGACAGGCTCCACTAGGTC AGTATATT
oEST063	yqjH-cat-mut-for	TCTCCATCGcCGcAGGCTATATGGACATGACCGATACA
oEST070	yqjH-amplify-rev-iso	ctgagcgagggagcagaaggatccTCAGCTTTTCTTTTCA TCTTGAAA
oEST071	yqjH-downstream-for-iso	gtagttgaccagtgctccctggtcATCGCTTGAAAAAAAG GGTG
oEST072	yqjW-Nter-rev-iso	ctgagcgagggagcagaaggatccCACTTTTTCTTTCATC ATACACACC
oEST073	yqjW-Cter-for-iso	gtagttgaccagtgctccctggtcAAAATAGGGGGGCATT ATAAA

Table S2: *B. subtilis* bacterial strains used in this study

Number	Designation or description	Relevant genotype	Construction or source strain designation	Reference
EST003	*B. subtilis* prototrophic wild-type strain	PY79	—	(1, 2)
EST053	DnaX-mYPet	PY79 *dnaX-mYpet cat Ω pWX340a*	Gift of Xindan Wang (Indiana University); strain BWX519 from plasmid pWX340	(3)
EST057	PolC-mYPet	PY79 *polC-mYpet cat*	Gift of Xindan Wang (Indiana University); strain BWX499	—
EST081	PolC-Dendra2	PY79 *polC-dendra2 loxP spec*	Gift of Xindan Wang (Indiana University); strain BWX2913	—
EST083	PolC-HaloTag	PY79 *polC-halo loxP spec*	Gift of Joseph Loparo (Harvard Medical School); strain ET096	—
EST111	ΔPol Y1	PY79 *yqjH::loxP spec*	Transformation: *yqjH::loxP spec* → EST003	This study
EST115	Pol Y1 loxP spec	PY79 *yqjH loxP spec*	Transformation: *yqjH loxP spec* → EST003	This study
EST117	ΔPol Y2	PY79 *yqjW::loxP spec*	Transformation: *yqjW::loxP spec* → EST003	This study
EST119	Pol Y1-CBM-mut1	PY79 *yqjH-CBM-mut1 loxP spec*	Transformation: *yqjH-CBM-mut1 loxP spec* → EST003	This study
EST121	Pol Y1-CBM-mut2	PY79 *yqjH-CBM-mut2 loxP spec*	Transformation: *yqjH-CBM-mut2 loxP spec* → EST003	This study
EST139	Pol Y1-Dendra2	PY79 *yqjH-dendra2 loxP spec*	Transformation: *yqjH-dendra2 loxP spec* → EST003	This study
EST141	Pol Y1-mYPet	PY79 *yqjH-mYPet loxP spec*	Transformation: *yqjH-mYPet loxP spec* → EST003	This study
EST143	Pol Y1-Halo	PY79 *yqjH-halo loxP spec*	Transformation: *yqjH-halo loxP spec* → EST003	This study
EST191	Pol Y1-cat-mut	PY79 *yqjH-cat-mut loxP spec*	Transformation: *yqjH-cat-mut loxP spec* → EST003	This study
EST197	Pol Y1-Halo DnaX-mYPet	PY79 *yqjH-halo loxP spec dnaX-mYpet cat Ω pWX340a*	Transformation: EST143 → EST053	This study

EST215	Pol Y1-CBM-mut1-Halo	PY79 *yqjH-CBM-mut1-halo loxP spec*	Transformation: *yqjH-CBM-mut1-halo loxP spec* → EST003	This study
EST217	Pol Y1-CBM-mut2-Halo	PY79 *yqjH-CBM-mut2-halo loxP spec*	Transformation: *yqjH-CBM-mut2-halo loxP spec* → EST003	This study
EST219	Pol Y1-CBM-mut1-Halo DnaX-mYPet	PY79 *yqjH-CBM-mut1-halo loxP spec dnaX-mYpet cat Ω pWX340a*	Transformation: EST215 → EST053	This study
EST221	Pol Y1-CBM-mut2-Halo DnaX-mYPet	PY79 *yqjH-CBM-mut2-halo loxP spec dnaX-mYpet cat Ω pWX340a*	Transformation: EST217 → EST053	This study
EST229	Pol Y1-cat-mut-Halo	PY79 *yqjH-cat-mut-halo loxP spec*	Transformation: *yqjH-cat-mut-halo loxP spec* → EST003	This study
EST243	Pol Y1-cat-mut-Halo DnaX-mYPet	PY79 *yqjH-cat-mut-halo loxP spec dnaX-mYpet cat Ω pWX340a*	Transformation: EST229 → EST053	This study
EST273	HBsu-mYPet	PY79 *sacA::hbsu-mYpet b.s. cat*	Gift of Xindan Wang (Indiana University); strain BWX583	(3)
EST275	Pol Y1-Halo HBsu-mYPet	PY79 *yqjH-halo b.s. loxP spec sacA::hbsu-mYpet b.s. cat*	Transformation: EST143 → EST273	This study

Table S3: Imaging dataset size

Dataset/Condition	Figure(s)	Number of Days	Number of Replicates	Number of Cells	Number of Tracks or Foci
DnaX cellular localization<br>Untreated	3B, S2C	8	8	816	1,363
WT Pol Y1 cellular localization<br>Untreated	3C, S2A	8	8	816	26,602
DnaX cellular localization<br>10 μM 4-NQO (1 h)	3D	4	6	760	1,522
WT Pol Y1 cellular localization<br>10 μM 4-NQO (1 h)	3E, S2B	4	6	760	40,469
WT Pol Y1-DnaX $g(r)$<br>Untreated (All trajectories)	4A, 4C, S5C	8	8	816	25,009
WT Pol Y1-DnaX $g(r)$<br>Untreated (Static: $D^* < 0.14\ \mu m^2/s$)	4A	8	8	816	8,465
WT Pol Y1-DnaX $g(r)$<br>Untreated (Mobile: $D^* > 0.14\ \mu m^2/s$)	4A	8	8	816	16,544
Pol Y1-CBM-mut1-DnaX $g(r)$<br>Untreated	4B	4	4	636	8,298
Pol Y1-CBM-mut2-DnaX $g(r)$<br>Untreated	4B	3	3	872	14,694
Pol Y1-cat-mut-DnaX $g(r)$<br>Untreated	4B	3	3	593	10,389
WT Pol Y1-DnaX $g(r)$<br>1 μM 4-NQO (1 h)	4C	3	4	621	9,357
WT Pol Y1-DnaX $g(r)$<br>10 μM 4-NQO (1 h)	4C, S5C	4	6	760	39,558
WT Pol Y1-DnaX $g(r)$<br>10 μM 4-NQO (2 h)	4C	2	3	238	16,728
WT Pol Y1 D^*<br>Untreated (All trajectories)	5A, 5H, S6A, S6J	8	8	816	24,544
WT Pol Y1 D^*<br>Untreated (Colocalized: < 200 nm to DnaX)	5B	8	8	816	4,856
WT Pol Y1 D^*<br>Untreated (Not colocalized: > 200 nm to DnaX-mYPet)	5C	8	8	816	18,264
WT Pol Y1 D^*<br>10 μM 4-NQO (1 h)	5D, 5H, S6C	4	6	760	37,383
Pol Y1-CBM-mut1 D^*<br>Untreated	5E	4	4	636	8,010
Pol Y1-CBM-mut2 D^*<br>Untreated	5F	3	3	872	13,953
Pol Y1-cat-mut D^*<br>Untreated	5G	3	3	593	10,085
WT Pol Y1 D^*<br>Fixed	S1B	2	2	282	3,364
WT Pol Y1 Mean PSF Width<br>Fixed (Long exposure)	S1C	2	2	294	2,358
False positive spots cellular localization<br>Untreated	S2A	2	2	158	113

False positive spots cellular localization<br>10 μM 4-NQO (1 h)	S2B	2	2	268	310
DnaX cellular localization<br>Untreated and unlabeled	S2D	2	2	267	441
WT Pol Y1 cellular localization and HBsu nucleoid profile<br>Untreated (All trajectories)	S2E	2	2	366	3,210
WT Pol Y1 cellular localization and HBsu nucleoid profile<br>Untreated (Static: $D^* < 0.14\ \mu m^2/s$)	S2F	2	2	366	1,399
DnaX cellular localization<br>Untreated (Long exposure)	S3A	8	8	916	1,544
DnaX cellular localization<br>10 μM 4-NQO (1 h) (Long exposure)	S3B	4	6	820	1,668
WT Pol Y1 cellular localization<br>Untreated (Long exposure)	S3C	8	8	916	19,538
WT Pol Y1 cellular localization<br>10 μM 4-NQO (1 h) (Long exposure)	S3D	4	6	820	21,905
WT Pol Y1 cellular localization<br>1 μM 4-NQO (1 h)	S3E	3	4	621	9,732
WT Pol Y1 cellular localization<br>10 μM 4-NQO (2 h)	S3F	2	3	238	17,142
Pol Y1-CBM-mut1 cellular localization<br>Untreated	S4A	4	4	636	8,933
Pol Y1-CBM-mut1 cellular localization<br>10 μM 4-NQO (1 h)	S4B	2	3	600	18,634
Pol Y1-CBM-mut2 cellular localization<br>Untreated	S4C	3	3	872	15,382
Pol Y1-CBM-mut2 cellular localization<br>10 μM 4-NQO (1 h)	S4D	2	3	697	21,630
Pol Y1-cat-mut cellular localization<br>Untreated	S4E	3	3	593	10,944
Pol Y1-cat-mut cellular localization<br>10 μM 4-NQO (1 h)	S4F	3	3	674	24,904
WT Pol Y1-DnaX $g(r)$<br>Untreated (Long exposure)	S5B	8	8	916	18,545
WT Pol Y1-DnaX $g(r)$<br>10 μM 4-NQO (1 h) (Long exposure)	S5B	4	6	820	21,249
Pol Y1-CBM-mut1-DnaX $g(r)$<br>10 μM 4-NQO (1 h)	S5C	2	3	600	18,170
Pol Y1-CBM-mut2-DnaX $g(r)$<br>10 μM 4-NQO (1 h)	S5C	2	3	697	20,573
Pol Y1-cat-mut-DnaX $g(r)$<br>10 μM 4-NQO (1 h)	S5C	3	3	674	24,296
False positive spots D^*<br>Untreated	S6B	2	2	158	98
False positive spots D^*<br>10 μM 4-NQO (1 h)	S6D	2	2	268	277
WT Pol Y1 D^*<br>1 μM 4-NQO (1 h)	S6E	3	4	621	9,029
WT Pol Y1 D^*<br>10 μM 4-NQO (2 h)	S6F	2	3	238	15,976
Pol Y1-CBM-mut1 D^*<br>10 μM 4-NQO (1 h)	S6G	2	3	600	16,892

Pol Y1-CBM-mut2 D^*<br>10 μM 4-NQO (1 h)	S6H	2	3	697	19,615
Pol Y1-cat-mut D^*<br>10 μM 4-NQO (1 h)	S6I	3	3	674	23,101
WT Pol Y1 jump lengths<br>Untreated	S7A	8	8	816	92,632
WT Pol Y1 jump lengths<br>1 μM 4-NQO (1 h)	S7B	3	4	621	33,844
WT Pol Y1 jump lengths<br>10 μM 4-NQO (1 h)	S7C	4	6	760	131,239
WT Pol Y1 jump lengths<br>10 μM 4-NQO (2 h)	S7D	2	3	238	51,372
Pol Y1-CBM-mut1 jump lengths<br>Untreated	S7E	4	4	636	41,042
Pol Y1-CBM-mut2 jump lengths<br>Untreated	S7F	3	3	872	62,925
Pol Y1-cat-mut jump lengths<br>Untreated	S7G	3	3	593	39,260
Pol Y1-CBM-mut1 jump lengths<br>10 μM 4-NQO (1 h)	S7H	2	3	600	75,690
Pol Y1-CBM-mut2 jump lengths<br>10 μM 4-NQO (1 h)	S7I	2	3	697	85,385
Pol Y1-cat-mut jump lengths<br>10 μM 4-NQO (1 h)	S7J	3	3	674	77,323
WT Pol Y1 binding lifetime<br>Untreated	S8B,<br>S8C,<br>S8E	8	8	916	19,538
WT Pol Y1 binding lifetime<br>Fixed	S8B,<br>S8C,<br>S8D	2	2	294	6,207
Pol Y1-cat-mut binding lifetime<br>Untreated	S8B, S8F	3	3	645	13,235
WT Pol Y1 binding lifetime<br>10 μM 4-NQO (1 h)	S8C,<br>S8G	4	6	820	21,905

Table S4: Value of the mean radial distribution function *g*(*r*) for Pol Y1-DnaX colocalization at the second smallest value of *r* (generally the maximum of the *g*(*r*) curve) and the standard error of the mean (S.E.M.) at that *r* value for the 100 calculated *g*(*r*) curves

**Figure(s)**	**Protein**	**Condition**	***g*(*r*) ± S.E.M.**
4A, 4C	WT Pol Y1	Untreated<br>(All trajectories)	2.84 ± 0.01
4A	WT Pol Y1	Untreated<br>(Static: D^* < 0.14 μm²/s)	5.95 ± 0.04
	WT Pol Y1	Untreated<br>(Mobile: D^* > 0.14 μm²/s)	1.112 ± 0.006
4B	Pol Y1-CBM-mut1	Untreated	0.157 ± 0.001
	Pol Y1-CBM-mut2	Untreated	0.0938 ± 0.0005
	Pol Y1-cat-mut	Untreated	3.20 ± 0.02
4C, S5C	WT Pol Y1	10 μM 4-NQO (1 h)	2.370 ± 0.007
4C	WT Pol Y1	1 μM 4-NQO (1 h)	2.14 ± 0.02
	WT Pol Y1	10 μM 4-NQO (2 h)	2.54 ± 0.01
S5B	WT Pol Y1	Untreated; Long exposure	2.76 ± 0.01
	WT Pol Y1	10 μM 4-NQO (1 h); Long exposure	2.79 ± 0.01
S5C	Pol Y1-CBM-mut1	10 μM 4-NQO (1 h)	0.536 ± 0.003
	Pol Y1-CBM-mut2	10 μM 4-NQO (1 h)	0.435 ± 0.002
	Pol Y1-cat-mut	10 μM 4-NQO (1 h)	3.05 ± 0.01

Table S5: Pol Y1-Halo diffusion coefficient distribution fit parameters from MSD analysis (± uncertainties from 95% fit confidence intervals)

Strain/Condition	D_1 (μm²/s)	A_1	D_2 (μm²/s)	A_2	D_3 (μm²/s)	A_3
WT Pol Y1 Untreated (All)	0.080 ± 0.005	0.281 ± 0.028	0.976 ± 0.077	0.468 ± 0.030	0.234 ± 0.030	0.250 ± 0.058
WT Pol Y1 Untreated (< 200 nm to DnaX-mYPet)	0.074 ± 0.006	0.440 ± 0.069	0.734 ± 0.187	0.209 ± 0.046	0.186 ± 0.032	0.351 ± 0.256
WT Pol Y1 Untreated (> 200 nm to DnaX-mYPet)	0.084 ± 0.005	0.243 ± 0.0234	0.996 ± 0.065	0.522 ± 0.028	0.253 ± 0.031	0.235 ± 0.051
WT Pol Y1 10 μM 4-NQO (1 h)	0.079 ± 0.004	0.282 ± 0.026	1.022 ± 0.092	0.459 ± 0.034	0.253 ± 0.032	0.259 ± 0.060
Pol Y1-CBM-mut1 Untreated	0.081 ± 0.005	0.108 ± 0.010	1.117 ± 0.041	0.682 ± 0.023	0.325 ± 0.028	0.210 ± 0.033
Pol Y1-CBM-mut2 Untreated	0.086 ± 0.005	0.086 ± 0.008	1.159 ± 0.031	0.725 ± 0.018	0.344 ± 0.025	0.188 ± 0.026
Pol Y1-cat-mut Untreated	0.079 ± 0.005	0.336 ± 0.038	1.009 ± 0.085	0.427 ± 0.026	0.204 ± 0.029	0.237 ± 0.064
WT Pol Y1 1 μM 4-NQO (1 h)	0.079 ± 0.005	0.314 ± 0.033	1.047 ± 0.085	0.474 ± 0.028	0.220 ± 0.034	0.213 ± 0.061
WT Pol Y1 10 μM 4-NQO (2 h)	0.081 ± 0.004	0.280 ± 0.027	0.994 ± 0.072	0.461 ± 0.026	0.230 ± 0.026	0.260 ± 0.053
Pol Y1-CBM-mut1 10 μM 4-NQO (1 h)	0.084 ± 0.005	0.118 ± 0.010	1.136 ± 0.041	0.687 ± 0.024	0.342 ± 0.032	0.195 ± 0.034
Pol Y1-CBM-mut2 10 μM 4-NQO (1 h)	0.076 ± 0.005	0.110 ± 0.009	1.128 ± 0.039	0.722 ± 0.023	0.332 ± 0.035	0.168 ± 0.032
Pol Y1-cat-mut 10 μM 4-NQO (1 h)	0.077 ± 0.005	0.357 ± 0.042	1.053 ± 0.120	0.423 ± 0.034	0.212 ± 0.039	0.220 ± 0.076

Table S6: Pol Y1-Halo diffusion coefficients and populations from Spot-On analysis

Strain/Condition	D_{static} (μm^2/s)	A_{static}	D_{free} (μm^2/s)	A_{free}
WT Pol Y1<br>Untreated (All)	0.024	0.401	0.880	0.599
WT Pol Y1<br>10 μM 4-NQO (1 h)	0.023	0.407	0.891	0.593
Pol Y1-CBM-mut1<br>Untreated	0.058	0.190	0.989	0.810
Pol Y1-CBM-mut2<br>Untreated	0.076	0.166	1.020	0.834
Pol Y1-cat-mut<br>Untreated	0.018	0.447	0.943	0.553
WT Pol Y1<br>1 μM 4-NQO (1 h)	0.019	0.404	0.949	0.596
WT Pol Y1<br>10 μM 4-NQO (2 h)	0.027	0.419	0.923	0.581
Pol Y1-CBM-mut1<br>10 μM 4-NQO (1 h)	0.049	0.180	0.998	0.820
Pol Y1-CBM-mut2<br>10 μM 4-NQO (1 h)	0.041	0.170	1.006	0.830
Pol Y1-cat-mut<br>10 μM 4-NQO (1 h)	0.014	0.470	0.952	0.530

Table S7: Photobleaching-corrected Pol Y1-Halo binding lifetimes (± uncertainties from 95% fit confidence intervals)

Strain/Condition	τ_{bleach} (s)	τ_{app} (s)	τ_{bound} (s)
WT Pol Y1<br>Fixed	1.10 ± 0.05	—	—
WT Pol Y1<br>Untreated	—	0.70 ± 0.02	1.9 ± 0.4
Pol Y1-cat-mut<br>Untreated	—	0.92 ± 0.03	6 ± 3
WT Pol Y1<br>10 μM 4-NQO (1 h)	—	0.73 ± 0.02	2.2 ± 0.5

Table S8: Fold change in number of colony forming units per mL (CFUs/mL) for imaging cultures after different treatments (mean ± standard deviation)

Condition	Untreated	DMF Only	1 μM 4-NQO (1 h)	10 μM 4-NQO (1 h)	10 μM 4-NQO (2 h)	50 μM 4-NQO (1 h)
**Fold Change in CFUs/mL**	2.09 ± 0.08	2.3 ± 0.6	2.1 ± 0.2	0.7 ± 0.2	1.5 ± 0.3	0.06 ± 0.02

Table S9: Mutagenesis for different strains in untreated or 4-NQO-treated cells measured by the rate of rifampicin resistance (Rif^R) and the induced mutation rate (4-NQO-treated mutation rate – untreated mutation rate) (mean ± standard deviation)

**Strain**	**WT**		**ΔPol Y1**		**ΔPol Y2**	
**Condition**	**Untreated**	**10 μM 4-NQO (1 h)**	**Untreated**	**10 μM 4-NQO (1 h)**	**Untreated**	**10 μM 4-NQO (1 h)**
**Rif^R (per 10^8)**	1.4 ± 0.4	1.9 ± 0.5	1.5 ± 0.5	2.4 ± 0.7	1.3 ± 0.4	2.0 ± 0.3
**Induced Rif^R (per 10^8)**	0.5 ± 0.3		1.0 ± 1.0		0.7 ± 0.4	

Supplementary Methods

Overview of strain construction strategy:

Bacterial strains containing fluorescent protein fusions or other modifications were constructed by transformation of either double-stranded DNA (dsDNA) fragments or genomic DNA bearing 1 – 2 kilobase (kb) homology arms for incorporation into the chromosome. dsDNA fragments containing desired modifications were synthesized by polymerase chain reaction (PCR) amplification and Gibson assembly.(4) Genomic DNA was extracted with phenol-chloroform and ethanol precipitation, resuspended in Tris-EDTA (TE) buffer, and transformed without further purification. Recipient strains were grown at 37 °C in BMK Complete medium (60 mM K_2HPO_4, 38 mM KH_2PO_4, 111 mM D-glucose, 3 mM sodium citrate, 0.0022% ferric ammonium citrate, 15 mM L-aspartic acid potassium salt, 10 mM $MgSO_4$, 0.05% yeast extract) to induce competence. Transformants were selected on LB Lennox agar plates containing the appropriate antibiotic, followed by one round of streak purification after the initial selection step. Newly transformed modifications were validated by PCR amplification of genomic DNA followed by Sanger DNA sequencing. Preexisting modifications were checked by streaking on antibiotic plates. Antibiotic concentrations used were 100 μg/mL spectinomycin and 5 μg/mL chloramphenicol. Tables S1 and S2 list all oligonucleotides and bacterial strains used in this study. Construction details for all new strains are summarized below.

The Pol Y1-Halo construct for microscopy was designed as a C-terminal fusion to the self-labeling HaloTag(5) with an 11 amino acid linker (GSGQGSGPGSG) between the Pol Y1 C-terminus and the HaloTag N-terminus. Similar C-terminal Pol Y1 fusions were made to the monomeric YFP variant mYPet(6) (using the 8 amino acid linker LEGSGQGP) and the photoconvertible green fluorescent protein (GFP) variant Dendra2(7, 8) (using the 8 amino acid linker LEGSGGSG). The DnaX replisome marker contained a C-terminal fusion to mYPet with an 8 amino acid linker (LEGSGQGP). The Pol Y1 catalytically inactive mutant contained the D108A E109A mutation to the catalytic residues in the polymerase active site.(9) The Pol Y1 CBM-mut1 and CBM-mut2 mutants contained mutations (QADAF and ALDLA respectively) to the WT clamp-binding motif sequence (QLDLF).

The *yqjW* gene is the first gene in an operon.(10) To minimize effects on the expression of the downstream gene, *yqjX*, the N-terminal 18 base pairs (bp) and the C-terminal 24 bp of *yqjW* (including the stop codon) were retained in the *yqjW::spec* knockout strain.

Detailed strain construction information:

EST111: ΔPol Y1. The *yqjH* upstream was amplified from strain EST003 using oligonucleotides oEST037 and oEST038. The loxP spec cassette was amplified from strain EST081 using oligonucleotides oEST039 and oEST040. The *yqjH* downstream was amplified from strain

EST003 using oligonucleotides oEST041 and oEST028. The three fragments were joined by Gibson assembly and transformed into strain EST003.

EST115: Pol Y1 loxP spec. The *yqjH* gene and upstream were amplified from strain EST003 using oligonucleotides oEST023 and oEST070. The loxP spec cassette was amplified from strain EST081 using oligonucleotides oEST032 and oEST008. The *yqjH* downstream was amplified from strain EST003 using oligonucleotides oEST071 and oEST028. The three fragments were joined by Gibson assembly and transformed into strain EST003.

EST117: ΔPol Y2. The *yqjW* upstream was amplified from strain EST003 using oligonucleotides oEST049 and oEST072. The loxP spec cassette was amplified from strain EST081 using oligonucleotides oEST032 and oEST008. The *yqjW* downstream was amplified from strain EST003 using oligonucleotides oEST073 and oEST048. The three fragments were joined by Gibson assembly and transformed into strain EST003.

EST119: Pol Y1-CBM-mut1. The N-terminal portion of *yqjH* (from the N-terminus to the clamp-binding motif) and the upstream were amplified from strain EST115 using oligonucleotides oEST023 and oEST058. The C-terminal portion of *yqjH* (from the clamp-binding motif to the C-terminus) and the downstream were amplified from strain EST115 using oligonucleotides oEST059 and oEST028. Oligonucleotides oEST058 and oEST059 contained the CBM-mut1 mutations. The two fragments were joined by Gibson assembly and transformed into strain EST003.

EST121: Pol Y1-CBM-mut2. The N-terminal portion of *yqjH* (from the N-terminus to the clamp-binding motif) and the upstream were amplified from strain EST115 using oligonucleotides oEST023 and oEST060. The C-terminal portion of *yqjH* (from the clamp-binding motif to the C-terminus) and the downstream were amplified from strain EST115 using oligonucleotides oEST061 and oEST028. Oligonucleotides oEST060 and oEST061 contained the CBM-mut2 mutations. The two fragments were joined by Gibson assembly and transformed into strain EST003.

EST139: Pol Y1-Dendra2. The *yqjH* gene and upstream were amplified from strain EST003 using oligonucleotides oEST023 and oEST029. The linker and Dendra2 were amplified from strain EST081 using oligonucleotides oEST033 and oEST034. The loxP spec cassette and *yqjH* downstream were amplified from strain EST115 using oligonucleotides oEST032 and oEST028. The three fragments were joined by Gibson assembly and transformed into strain EST003.

EST141: Pol Y1-mYPet. The *yqjH* gene and upstream were amplified from strain EST003 using oligonucleotides oEST023 and oEST029. The linker and mYPet were amplified from strain EST057 using oligonucleotides oEST035 and oEST036. The loxP spec cassette and *yqjH*

downstream were amplified from strain EST115 using oligonucleotides oEST032 and oEST028. The three fragments were joined by Gibson assembly and transformed into strain EST003.

EST143: Pol Y1-Halo. The *yqjH* gene and upstream were amplified from strain EST003 using oligonucleotides oEST023 and oEST029. The linker and HaloTag were amplified from strain EST083 using oligonucleotides oEST030 and oEST031. The loxP spec cassette and *yqjH* downstream were amplified from strain EST115 using oligonucleotides oEST032 and oEST028. The three fragments were joined by Gibson assembly and transformed into strain EST003.

EST191: Pol Y1-cat-mut. The N-terminal portion of *yqjH* (from the N-terminus to the catalytic site) and the upstream were amplified from strain EST115 using oligonucleotides oEST037 and oEST062. The C-terminal portion of *yqjH* (from the clamp-binding motif to the C-terminus) and the downstream were amplified from strain EST115 using oligonucleotides oEST063 and oEST028. Oligonucleotides oEST062 and oEST063 contained the catalytic site mutations. The two fragments were joined by Gibson assembly and transformed into strain EST003.

EST197: Pol Y1-Halo DnaX-mYPet. The *yqjH-halo loxP spec* allele was transferred from strain EST143 to strain EST053 by transformation with genomic DNA.

EST215: Pol Y1-CBM-mut1-Halo. The *yqjH* gene (containing the CBM-mut1 mutation) and upstream were amplified from strain EST119 using oligonucleotides oEST023 and oEST029. The linker and HaloTag were amplified from strain EST083 using oligonucleotides oEST030 and oEST031. The loxP spec cassette and *yqjH* downstream were amplified from strain EST115 using oligonucleotides oEST032 and oEST028. The three fragments were joined by Gibson assembly and transformed into strain EST003.

EST217: Pol Y1-CBM-mut2-Halo. The *yqjH* gene (containing the CBM-mut2 mutation) and upstream were amplified from strain EST121 using oligonucleotides oEST023 and oEST029. The linker and HaloTag were amplified from strain EST083 using oligonucleotides oEST030 and oEST031. The loxP spec cassette and *yqjH* downstream were amplified from strain EST115 using oligonucleotides oEST032 and oEST028. The three fragments were joined by Gibson assembly and transformed into strain EST003.

EST219: Pol Y1-CBM-mut1-Halo DnaX-mYPet. The *yqjH-CBM-mut1-halo loxP spec* allele was transferred from strain EST215 to strain EST053 by transformation with genomic DNA.

EST221: Pol Y1-CBM-mut2-Halo DnaX-mYPet. The *yqjH-CBM-mut2-halo loxP spec* allele was transferred from strain EST217 to strain EST053 by transformation with genomic DNA.

EST229: Pol Y1-cat-mut-Halo. The *yqjH* gene (containing the catalytic site mutations) and upstream were amplified from strain EST191 using oligonucleotides oEST023 and oEST029. The linker and HaloTag were amplified from strain EST083 using oligonucleotides oEST030 and oEST031. The loxP spec cassette and *yqjH* downstream were amplified from strain EST115 using oligonucleotides oEST032 and oEST028. The three fragments were joined by Gibson assembly and transformed into strain EST003.

EST243: Pol Y1-cat-mut-Halo DnaX-mYPet. The *yqjH-cat-mut-halo loxP spec* allele was transferred from strain EST229 to strain EST053 by transformation with genomic DNA.

EST275: Pol Y1-Halo HBsu-mYPet. The *yqjH-halo loxP spec* allele was transferred from strain EST143 to strain EST273 by transformation with genomic DNA.

Supplementary References

1. Zeigler,D.R., Prágai,Z., Rodriguez,S., Chevreux,B., Muffler,A., Albert,T., Bai,R., Wyss,M. and Perkins,J.B. (2008) The Origins of 168, W23, and Other *Bacillus subtilis* Legacy Strains. *J Bacteriol*, **190**, 6983–6995.
2. Schroeder,J.W. and Simmons,L.A. (2013) Complete Genome Sequence of Bacillus subtilis Strain PY79. *Genome Announc*, **1**, e01085-13.
3. Wang,X., Montero Llopis,P. and Rudner,D.Z. (2014) Bacillus subtilis chromosome organization oscillates between two distinct patterns. *Proc. Natl. Acad. Sci. U.S.A.*, **111**, 12877–12882.
4. Gibson,D.G., Young,L., Chuang,R.-Y., Venter,J.C., Hutchison,C.A. and Smith,H.O. (2009) Enzymatic assembly of DNA molecules up to several hundred kilobases. *Nat Methods*, **6**, 343–345.
5. Los,G.V., Encell,L.P., McDougall,M.G., Hartzell,D.D., Karassina,N., Zimprich,C., Wood,M.G., Learish,R., Ohana,R.F., Urh,M., *et al.* (2008) HaloTag: A Novel Protein Labeling Technology for Cell Imaging and Protein Analysis. *ACS Chem. Biol.*, **3**, 373–382.
6. Nguyen,A.W. and Daugherty,P.S. (2005) Evolutionary optimization of fluorescent proteins for intracellular FRET. *Nat. Biotechnol.*, **23**, 355–360.
7. Gurskaya,N.G., Verkhusha,V.V., Shcheglov,A.S., Staroverov,D.B., Chepurnykh,T.V., Fradkov,A.F., Lukyanov,S. and Lukyanov,K.A. (2006) Engineering of a monomeric green-to-red photoactivatable fluorescent protein induced by blue light. *Nat Biotechnol*, **24**, 461–465.
8. Chudakov,D.M., Lukyanov,S. and Lukyanov,K.A. (2007) Tracking intracellular protein movements using photoswitchable fluorescent proteins PS-CFP2 and Dendra2. *Nat Protoc*, **2**, 2024–2032.
9. Duigou,S., Ehrlich,S.D., Noirot,P. and Noirot-Gros,M.-F. (2004) Distinctive genetic features exhibited by the Y-family DNA polymerases in Bacillus subtilis. *Mol. Microbiol.*, **54**, 439–451.

10. Au,N., Kuester-Schoeck,E., Mandava,V., Bothwell,L.E., Canny,S.P., Chachu,K., Colavito,S.A., Fuller,S.N., Groban,E.S., Hensley,L.A., *et al.* (2005) Genetic Composition of the Bacillus subtilis SOS System. *J. Bacteriol.*, **187**, 7655–7666.
